# Convergent recombination suppression suggests role of sexual selection in guppy sex chromosome formation

**DOI:** 10.1038/ncomms14251

**Published:** 2017-01-31

**Authors:** Alison E. Wright, Iulia Darolti, Natasha I. Bloch, Vicencio Oostra, Ben Sandkam, Severine D. Buechel, Niclas Kolm, Felix Breden, Beatriz Vicoso, Judith E. Mank

**Affiliations:** 1 Department of Genetics, Evolution and Environment, University College London, Darwin Building, Gower Street, London WC1E 6BT, UK; 2 Department of Biological Sciences, Simon Fraser University, 8888 University Drive, Burnaby, British Columbia, Canada V5A 1S6; 3 Department of Zoology, Stockholm University, Svante Arrheniusväg 18 B, Stockholm 106 91, Sweden; 4 Institute of Science and Technology, Am Campus 1A, Klosterneuburg 3400, Austria

## Abstract

Sex chromosomes evolve once recombination is halted between a homologous pair of chromosomes. The dominant model of sex chromosome evolution posits that recombination is suppressed between emerging X and Y chromosomes in order to resolve sexual conflict. Here we test this model using whole genome and transcriptome resequencing data in the guppy, a model for sexual selection with many Y-linked colour traits. We show that although the nascent Y chromosome encompasses nearly half of the linkage group, there has been no perceptible degradation of Y chromosome gene content or activity. Using replicate wild populations with differing levels of sexually antagonistic selection for colour, we also show that sexual selection leads to greater expansion of the non-recombining region and increased Y chromosome divergence. These results provide empirical support for longstanding models of sex chromosome catalysis, and suggest an important role for sexual selection and sexual conflict in genome evolution.

## Abstract (web summary)

It has been suggested that sex chromosomes arise as a result of sexual conflict, resulting in selection against recombination between chromosomes. Here, the authors resequence laboratory and wild guppy populations with differing levels of sexual antagonism, providing support for this long-held view.

Sex chromosomes are typically thought to evolve as recombination is halted between a homologous pair of chromosomes in one sex. Although we have a detailed understanding of the evolutionary consequences of the loss of recombination for sex chromosome evolution^1^,^2^, we still do not understand the evolutionary forces acting to halt recombination in the first place. The dominant theoretical model for the early stages of sex chromosome evolution^3^,^4^,^5^ predicts that recombination will be selected against in the region between a sex determining gene and a nearby locus with alleles of sex-specific effect. This theory, though prevalent, remains largely untested empirically, as most research has focused on older, highly divergent sex chromosome systems^6^,^7^, for which it is difficult to extrapolate the earliest stages and causes of divergence.

The sex chromosomes of the guppy (*Poecilia reticulata*) have been of interest for more than a century, following early reports that many sexually selected colour traits are passed through the patriline on the Y chromosome^8^,^9^. These observations were central to the development of theories regarding the role of sexual conflict in recombination suppression and sex chromosome divergence^3^,^4^,^5^. Colour is sexually antagonistic in guppies, as brightly coloured males are more attractive to females and more visible to predators, but brightly coloured females gain no fitness advantage and only suffer increased predation^10^,^11^,^12^. Therefore, in this system, current models of sex chromosome evolution predict that recombination would be selected against between the sex determining locus and linked loci involved in colouration. This process would shrink the pseudoautosomal region in favour of expanding X- and Y-specific regions, creating a male supergene on the Y chromosome containing multiple colouration loci and thereby resolving sexually antagonistic selection.

Even though the guppy sex chromosomes are a classic model for the study of sexual conflict and sex chromosome divergence, little is actually known about the pattern of divergence between the X and Y chromosomes. Recent linkage maps identified male recombination events restricted to the middle of chromosome 12 (ref. 13), suggesting that the other half of the chromosome is functionally X- or Y-linked. Immunostaining of recombination nodules^14^ was broadly concordant with recombination mapping, again suggesting that the X chromosome is split roughly in equal parts between X-specific and pseudoautosomal.

Recombination shows substantial local variation between males and females throughout the genomes of many organisms^7^,^15^ and identifying areas of restricted male recombination does not distinguish the sex chromosome from other areas where males simply do not recombine. However, the Y is morphologically distinguishable from the X chromosome^16^, and comparative genome hybridization of lab populations^17^ suggest that roughly half of the Y chromosome is male-specific. Because many vertebrate sex chromosomes show progressive spread of the non-recombining region^18^,^19^,^20^,^21^, the large size of the guppy non-recombining region and male-specific regions suggest substantial divergence between the X and Y.

Recombination suppression between the X and Y chromosomes results in complete linkage of the male-specific region of the Y. The loss of recombination in this region typically limits the role of adaptive evolution and leads to strong background selection and linkage effects, causing loss of functional polymorphism in coding sequence over time^1^. Roughly half of male colouration patterns are thought to be Y-linked^8^, and the remarkable diversity of male colour combinations implies an improbably large number of Y haplotypes maintained within populations for a sex chromosome system of at least intermediate age. Additionally, if recombination suppression really is driven by sexually antagonistic alleles^3^,^4^,^5^, then we might expect recent but rapid spread of recombination suppression shortly after the emergence of sexual preferences for colour. Although sexually selected traits exist in many Poeciliids, the vivid male colouration in *P. reticulata* is only shared by a few very close relatives^22^,^23^, therefore, the expansions of the male-limited Y chromosome to engulf colouration loci might have occurred very recently.

Moreover, the degree of male colouration, and, therefore, the degree of sexual conflict over colour, varies substantially based on predation pressures. Across watersheds, downstream populations are typically associated with higher predation and males are far less colourful than upstream populations^24^,^25^,^26^. Importantly, the proportion of colour patterns thought to be Y-linked varies between upstream and downstream populations^27^. The unusual gene content of the guppy sex chromosomes, therefore, makes it a uniquely powerful system for testing the role of sexual conflict and sexual selection in sex chromosome divergence.

In order to determine the degree of divergence between the X and Y chromosome in this species, we resequenced male and female genomes and transcriptomes of both laboratory and wild individuals. We find that the X and Y show sequence differentiation over nearly one half of the length of the chromosome, however, the divergence between the X and Y chromosome is remarkably subtle, indicating very low levels of divergence and likely recent origin of the sex chromosome. The large region of divergence is in contrast to reports of other nascent sex chromosome systems where the diverged region is highly restricted^28^,^29^,^30^. Despite this young age, we detect evidence of Faster-X evolution in this region. Most importantly, we find convergent patterns of greater sex chromosome divergence in upstream populations, which experience substantially elevated sexual selection and sexual conflict, compared with downstream populations. Our results suggest that recombination suppression between the X and Y spread quickly in the recent history of this sex chromosome system, possibly driven by the presence of sexually antagonistic alleles related to sexual selection.

## Results

### The structure of the guppy sex chromosomes

We first assembled the female genome using SOAPdenovo2, based on 480 million paired end reads from an outbred laboratory population. The assembly yielded 96,611 scaffolds, with an N50 of 11.3Kb and total length of 634.8 Mb, after a minimum length threshold of 1 Kb (Supplementary Tables 1–3). Guppy genes from the reference genome (Guppy_female_1.0+MT) were mapped to scaffolds in order to identify chromosomal positions, resulting in a final assembly of 19,206 ordered scaffolds oriented along the guppy chromosomes, with an N50 of 17.4 Kb and total length of 219.5 Mb (Supplementary Tables 3 and 4).

We then mapped male and female DNA-seq reads to our ordered scaffolds in order to identify regions of coverage difference between the sexes. Regions with longstanding recombination suppression in males will show reduced mapping efficiency against the female genome assembly, as diverged sequence from the Y will no longer map to the X chromosome^19^,^20^,^31^. Even with strict mapping thresholds (see methods) we could identify no large region of the genome with reduced coverage in males, which we would expect if a large portion of the Y was significantly diverged or degraded (Supplementary Fig. 1), and the overall distribution of coverage is largely symmetrical (Supplementary Fig. 2A). However, previous linkage maps have identified chromosome 12, which contains the sex determining gene, as the sex chromosome^13^, and this chromosome shows a slight shift in the distribution and has a significantly greater proportion of scaffolds with female-biased coverage than autosomes (Wilcoxon rank sum test *P*<0.001, Supplementary Fig. 2B, Supplementary Table 5). This suggests that recombination suppression between the X and Y chromosomes has led to very slight divergence between them.

**Figure 1:**
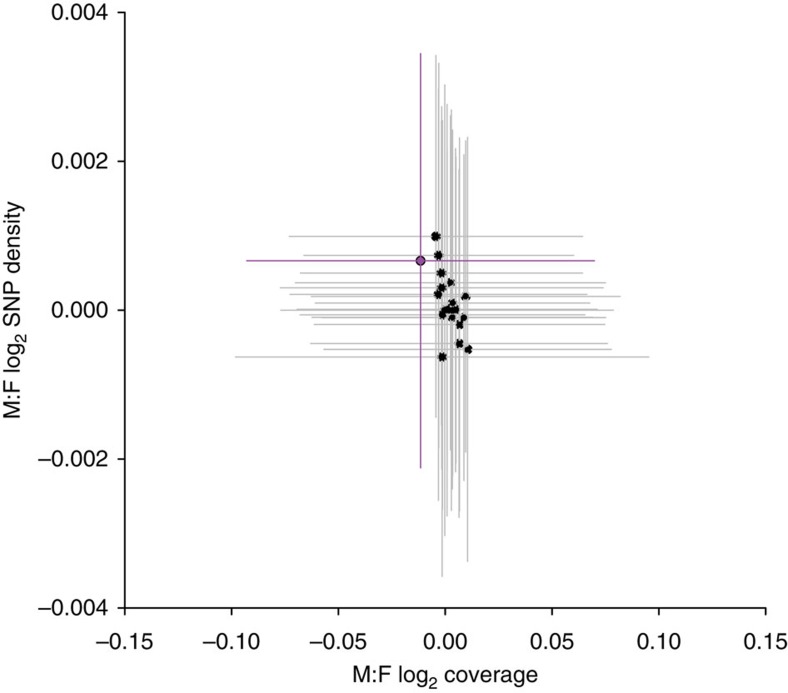
Distribution of sex differences in coverage and SNP density for all chromosomes. The X chromosome is in purple. Horizontal and vertical lines denote interquartile ranges.

If the Y has diverged, but not yet degraded significantly, we would expect to observe Y-specific single nucleotide polymorphisms (SNPs) in regions that retain substantial sequence similarity to the X, resulting in higher average male heterozygosity for the sex chromosomes^32^,^33^. When assessing all regions of the genome, we observe a shoulder of elevated SNP density in males (Supplementary Fig. 2C), due to significantly greater SNP density in males for the sex chromosomes compared with autosomal genes (Wilcoxon rank sum test *P*<0.001, Supplementary Fig. 2D, Supplementary Table 5). When sex differences in coverage and SNP density are plotted together, the sex chromosome is a clear outlier to the other chromosomes (Fig. 1), confirming low but significant levels of divergence.

**Figure 2:**
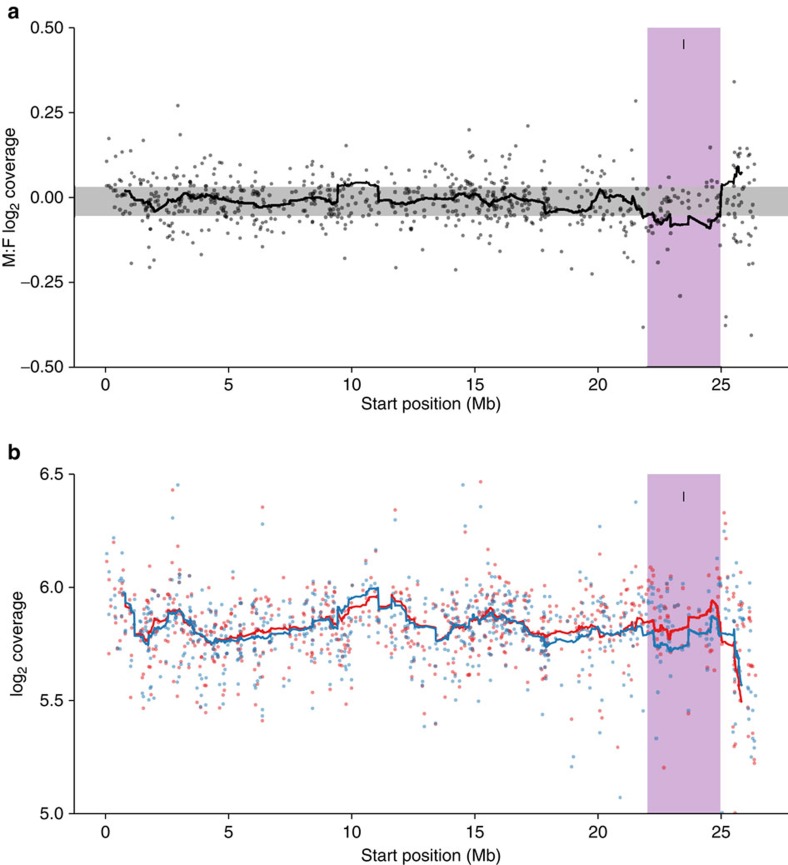
Male and female coverage characteristics of guppy sex chromosome. (**a**) Moving average of coverage differences between male and female reads based on sliding window analysis (window size of 40 scaffolds). Ninety-five per cent confidence intervals based on bootstrapping autosomal estimates are in grey. (**b**) Male (blue) and female (red) coverage for the X chromosome. For both panels, dark purple indicates the region of the sex chromosomes with the greatest X-Y sequence divergence, where coverage is significantly less in males (Stratum I, 22–25 Mb).

**Figure 3:**
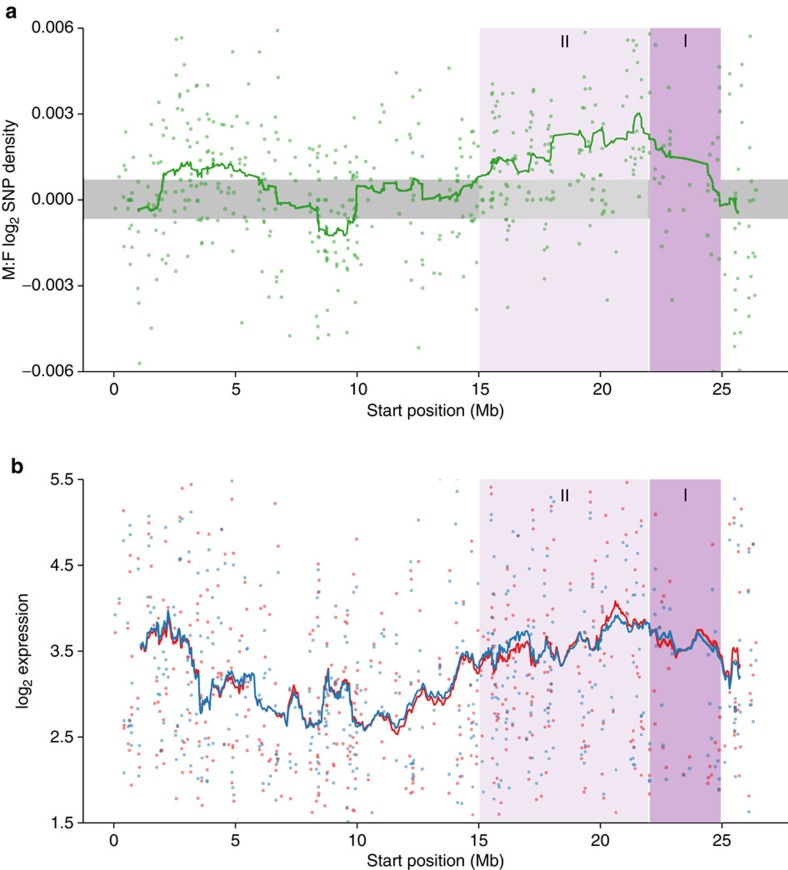
Male and female SNP density and expression differences on guppy sex chromosome. (**a**) Moving average of male:female SNP density based on sliding window analysis (window size of 40 scaffolds). Ninety-five per cent confidence intervals based on bootstrapping autosomal estimates are in grey. (**b**) Male (blue) and female (red) expression of genes along the X chromosome (window size of 40 genes). Dark purple indicates the region of the sex chromosomes with the greatest X-Y sequence divergence, where coverage is significantly less in males (Stratum I, 22–25 Mb) (see Fig. 2), light purple indicates the region with less X-Y differentiation, where there is a significant excess of male SNPs (Stratum II, 15–22 Mb).

In order to determine the relative divergence between X and Y chromosomes, we plotted coverage and SNP density differences between males and female on our scaffolds against physical position on the guppy genome assembly. We detected significantly reduced male coverage outside the autosomal 95% confidence interval from 22–25 Mb (Fig. 2). This region shows the largest degree of X-Y sequence divergence and likely corresponds to the oldest region of the sex chromosome (Stratum I). In contrast, between 15–25 Mb, we detect significant elevation of male SNP density but no reduction in male coverage, indicative of lower levels of X-Y divergence and suggesting that nearly half of the sex chromosome has stopped recombining in males in the very recent past (Stratum II) (Fig. 3a, Supplementary Fig. 3).

Our coverage and SNP analysis suggest that although male-specific SNPs have accumulated, the Y chromosome has not degenerated significantly. Because loss of gene activity often quickly follows loss of recombination on the Y chromosome^1^, for each gene we plotted male and female expression level (RPKM) across the X chromosome. Our results show that the non-recombining region exhibits low levels of sexualization of gene content, with regions where the majority of genes exhibit female- or male-biased expression. However, there is no region of detectible loss of male gene activity, as would be expected with extensive Y chromosome decay (Fig. 3b, Supplementary Fig. 4). In contrast, in the region of the sex chromosome with the greatest coverage difference between males and females (Stratum I, 22–25 Mb), likely the area of greatest Y chromosome divergence, there is a slight excess of male-biased genes, indicating that this region of the Y chromosome has also not suffered any significant loss of gene activity. We tested for enrichment of GO terms for genes expressed in the X-Y diverged region (Strata I and II, 15–25 Mb) relative to the rest of the genome. However, there were no GO terms with an enrichment *P*<0.001.

X chromosomes are predicted in many circumstances to show elevated rates of evolution^34^, and signatures of Faster-X evolution have been detected in old, heteromorphic sex chromosomes^35^,^36^,^37^. However, it is unclear whether a detectible signal of Fast-X would be expected in the early stages of sex chromosome evolution. We, therefore, compared rates of evolution for X-linked and autosomal coding sequence, and recovered a significant pattern of Faster-X in the guppy. X-linked *d*_N_/*d*_S_ is greater though marginally non-significant for X-linked genes (86 genes, permutation test with 1,000 replicates, *P*=0.067) relative to the autosomes (4,755 genes), due to a marginally significant increase in *d*_N_ (permutation test with 1000 replicates, *P*=0.014) (Supplementary Table 6, Supplementary Fig. 5). This pattern is evident across both Strata I and II, indicating that low levels of sex chromosome divergence are sufficient to facilitate Faster-X processes.

### Population variation in male colour and sexual conflict

Predation pressures vary substantially for natural guppy populations, with generally lower predation pressures upstream compared with downstream^26^. This has led to differences in female preference for male colouration^11^, with downstream males less vivid due to reduced female preferences and higher predation risks than upstream populations^24^,^25^,^38^. Upstream and downstream populations within watersheds are more closely related to each other than across watersheds (Supplementary Fig. 6A). Therefore, shifts in male colouration have occurred independently in each watershed^39^, where downstream males are less colourful than upstream males.

**Figure 4:**
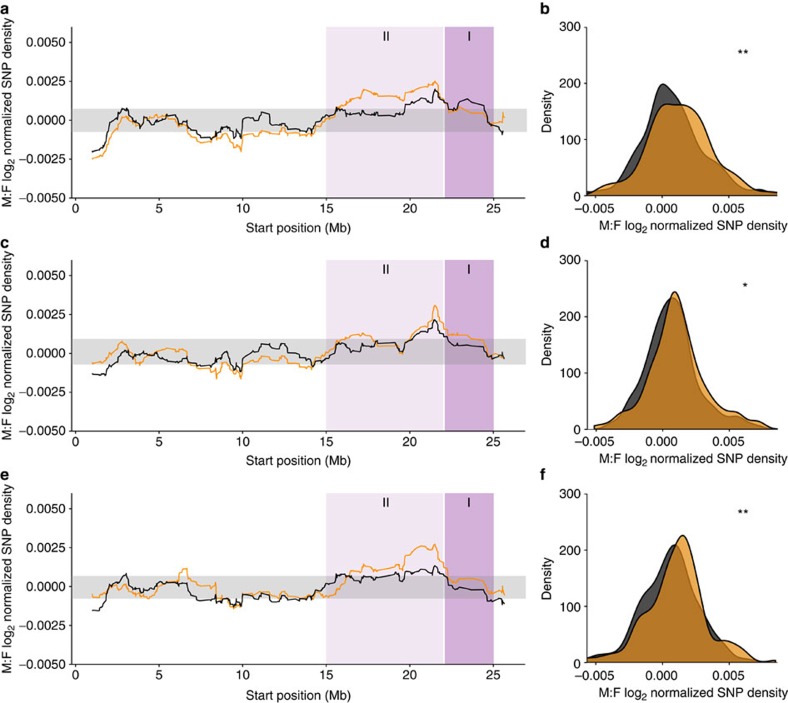
Male:female SNP density for the X chromosome across upstream (orange) and downstream (black) guppy populations. (**a**,**c** and **e**) Moving averages of normalized SNP density across the X chromosome based on sliding window analysis (window size of 40 genes) for Yarra (**a**), Quare (**c**) and Aripo (**e**) watersheds. Ninety-five per cent confidence intervals based on bootstrapping autosomal estimates are in grey. Dark purple indicates the region of the sex chromosomes with the greatest X-Y sequence divergence, where coverage is significantly less in laboratory population males (Stratum I, 22–25 Mb) (see Fig. 2), light purple indicates the region with less X-Y differentiation, where there is a significant excess of male SNPs in laboratory populations (Stratum II, 15–22 Mb) (see Fig. 3). (**b**,**d** and **f**) Distribution of sex differences in normalized SNP density for the X-Y diverged region (Strata I and II, 15–25 Mb) for Yarra (**b**), Quare (**d**) and Aripo (**f**) watersheds. **P-value<0.020, *P-value<0.050 based on permutation tests.

Given the very recent origin of the guppy sex chromosomes, we might expect that if recombination suppression is indeed driven by sexual conflict over colour, there might be differences in the divergence of the sex chromosomes across different populations with more or less male colouration. In line with this prediction, there is evidence that different populations of wild guppies display different patterns of Y-linkage of colour traits^40^. We, therefore, examined patterns of sex-specific heterozygosity for upstream and downstream populations of wild guppies. We sampled three watersheds (Yarra, Quare, Aripo) and from each watershed, four males were caught from an upstream population and four males were caught from a downstream population. Our results (Fig. 4, Supplementary Table 7) show that across replicate upstream populations, where males are more colourful, there is significantly greater divergence between the X and Y chromosomes than the ancestral downstream populations (Wilcoxon rank sum test between upstream and downstream populations across watersheds, Yarra *P*=0.011, Quare *P*=0.046, Aripo *P*=0.017). Expansion of the non-recombining region and corresponding X-Y divergence has occurred repeatedly and independently across populations, as the phylogeny of these populations reveals that in each watershed, upstream populations are consistently derived from downstream populations (Supplementary Fig. 6B). By randomly sampling 10 Mb windows with 1,000 repetitions across the autosomes, we find that the probability of observing this convergence in SNP density across populations by chance is *P*<0.004. In contrast, there are no differences in patterns of coverage between upstream and downstream populations in the area of greatest sex chromosome divergence (Stratum I, 22–25 Mb, Supplementary Fig. 7, Supplementary Table 8), indicating that X-Y divergence in this region predates the divergence of these wild populations.

## Discussion

Observations of Y-linkage for a large proportion of male colour patterns in guppies^8^,^9^ helped form the nucleus of theories regarding the role of sexual conflict in sex chromosome formation^3^,^4^,^5^. Here we used individuals from natural and laboratory populations in conjunction with analysis of coverage, SNP and expression differences between males and females in this model system to test the role of sexual conflict in recombination suppression between the X and Y chromosomes. Our results suggest two regions of divergence on the sex chromosome. One region, likely the area of greatest Y chromosome divergence, is manifest with slightly reduced DNA coverage in males in a restricted region spanning 22–25 Mb. A larger region of more recent recombination suppression from 15–22 Mb is distinguishable only through the build-up of Y-specific SNPs. In both regions, although male-specific SNPs have accumulated on the Y, there is no evidence of large-scale decay of the Y chromosome or loss of gene activity observed in many older sex chromosome systems^31^,^41^. Surprisingly, this region of divergence extends over nearly half of the sex chromosome, indicating that recombination has been suppressed over a large region very recently. The two strata we observe in guppies are consistent with step-wise patterns of sex chromosome formation observed in many other organisms, including mammals^42^, birds^21^, *Silene*^43^, sticklebacks^44^ and *Nothobranchius*^45^. In the latter case, the authors observed population-level variation in the youngest stratum, similar to what we observe in guppies, suggesting that strata can form independently within species.

Comparisons of coverage and SNP density between males and females, like the analyses we implement here, offer two complementary views of sex chromosome evolution. Coverage differences are expected in more diverged regions with significant Y chromosome degeneration. In contrast, sex-differences in SNP density, particularly in regions with elevated SNP density in the heterogametic sex, are expected in more diverged systems with little Y chromosome degeneration. However, implementing these approaches in young sex chromosome systems should be accompanied by information as to the location of the sex determining region, which has been previously mapped to the far end of chromosome 12 (ref. 13). Ideally, Y-specific sequence data would be useful in verifying and dating stratum boundaries. However, this is complicated in our system due to the lack of complete lineage sorting of Y-specific SNPs, precluding the reconstruction of Y-specific sequence from our short-read data. In future work, long read RNA-seq data, optical mapping and other phasing approaches will be useful in confirming stratum boundaries and identifying Y-linked sequences. These data will also be important in determining whether inversions, which are often assumed to be involved in recombination suppression, are indeed the mechanism behind sex chromosome divergence.

Despite the limited sequence divergence between the X and Y chromosomes, we observe two evolutionary signatures that are typically only associated with heteromorphic sex chromosome systems. First, the X chromosome shows the early stages of sexualization for gene expression despite limited evidence for degeneration in gene activity or content across the non-recombining Y chromosome. Previous evidence of sexualization comes from old, highly heteromorphic sex chromosome systems^46^,^47^,^48^ and it was previously unclear how quickly sex-biased expression can accumulate after sex chromosome formation. Our results, therefore, indicate that sexualization of the X chromosome can occur very quickly after recombination is halted. Second, we detect a Faster-X effect, where X-linked coding sequence diverges more rapidly than the remainder of the genome. Until now, evidence for Faster-X was restricted to highly diverged sex chromosomes^35^,^36^,^37^, however, our results suggest that the Faster-X processes can accumulate rapidly following the loss of recombination. These findings have important consequences for the role of sex chromosomes in Haldane's rule^49^ and the Large-X effect in speciation^50^, and suggests that young or undifferentiated sex chromosomes may act as an important driver in the evolution of reproductive isolation^51^.

Most systems where sex chromosomes have formed recently^28^,^29^,^30^, and even some older sex chromosome systems^20^,^52^, show restricted recombination in only a small region. The region of divergence extends over almost half of the sex chromosomes in the guppy, suggesting that recombination has been suppressed very quickly over a large region of the Y chromosome in guppies. This rapid spread of recombination suppression may have been driven by the presence of sexually antagonistic alleles related to male colour on the proto-sex chromosome^10^,^11^,^12^. The high proportion of Y-linked colour patterns in guppies^8^,^9^ is likely the product of rapid spread of recombination suppression between the X and Y chromosomes, which would resolve sexually antagonism by limiting colour expression to males.

Fish show remarkable variation in sex determination^6^,^53^ and rapid origin and turnover of sex chromosomes^6^,^54^. The tiger pufferfish has homomorphic sex chromosomes, where the sexes differ by only a single missense SNP^28^, whereas a significant proportion of the sex chromosomes in sticklebacks are non-recombining^44^,^55^, and there has been substantial decay of gene activity on the Y chromosome. Although studies in related species are required to date the exact age of the sex chromosomes in *P. reticulata*, there is extensive sex chromosome turnover in the poeciliid clade^54^,^56^, suggesting a recent origin of the sex chromosomes described here. This is consistent with expectations that the expansion of the Y-limited region was driven by sexual conflict over colouration, suggesting that Stratum II originated around the same period that male colouration emerged as a major component of female preference, likely <5Mya (refs 22, 23).

Our results suggest that this younger region of recombination suppression has expanded convergently in upstream populations as a consequence of increased sexual selection and sexual conflict over colouration^11^,^25^. We found the same convergent pattern of X and Y divergence between colourful upstream populations compared with the duller ancestral downstream populations over each of three replicate watersheds (Fig. 4). Upstream populations all showed greater divergence between the X and Y based on SNP density, and the region of significant SNP divergence extends over a larger region of the sex chromosomes. This accelerated divergence in upstream populations in each of the three watersheds has likely occurred independently, as populations within watersheds are well known to be monophyletic^39^. In support of this, our phylogenetic reconstruction reveals that in each watershed, upstream populations independently evolved from ancestral downstream populations. This suggests that sexual selection and sexual conflict over colour has driven greater Y divergence, consistent with longstanding theoretical predictions about the role of sexual antagonism in sex chromosome formation^3^,^4^,^5^. However, it is worth noting that our replicate upstream populations show some variation in the degree of differentiation, possibly due to demographic factors such as bottlenecks and recent expansions, date of colonization, rate of dispersal and gene flow between upstream and downstream populations, and effective population size, as well as stochastic processes.

Altogether, our results suggest that sexual conflict may be responsible for the remarkably rapid recent spread of recombination suppression to encompass colouration alleles within the Y chromosome. Moreover, our data are consistent with a role of sexual selection in accelerating divergence of the Y chromosome once recombination suppression is established.

## Methods

### Sample collection

All samples were collected in accordance with national and institutional ethical guidelines. First, we sampled males and females from a single large, outbred laboratory population established in 1998 (ref. 57). Tail samples were homogenized and stored in RNA later before RNA preparation, the remainder of each fish was stored in ethanol before DNA preparation.

Second, wild males were caught from three watersheds (Yarra, Quare, Aripo) in the Northern Range Mountains of Trinidad in February 2015 (see ref. 58 for description of the habitats). From each watershed, four males were caught from an upstream population and four males were caught from a downstream population. Samples were collected and stored immediately in ethanol prior to DNA preparation.

### Sequencing

Nucleic acids were extracted with RNAeasy Kit (Qiagen) and DNeasy Blood and Tissue Kit (Qiagen) using the manufacturer protocols. The libraries were prepared and barcoded at The Wellcome Trust Centre for Human Genetics, University of Oxford using standard protocols. RNA was sequenced on an Illumina HiSeq 2500 resulting in on average 32 million 100 bp paired-end reads per sample. DNA was sequenced on an Illumina HiSeq 4000, resulting in on average 269 million 100 bp paired-end reads per individual sampled from a single large, outbred laboratory population, and 123 million 100 bp paired-end reads per sample for individuals caught in the wild in Trinidad (Supplementary Table 1).

### Quality trimming and filtering

DNA data were quality assessed using FastQC v0.11.4 (www.bioinformatics.babraham.ac.uk/projects/fastqc) and quality trimmed using Trimmomatic v0.35 (ref. 59). We filtered reads containing adaptor sequences and trimmed reads if the sliding window average Phred score over four bases was <15 or if the leading/trailing bases had a Phred score <3. Reads were removed post filtering if either read pair was <50 bases in length. RNA-seq data was quality assessed and trimmed using the same criteria but with a minimum length threshold of 36 bases (Supplementary Table 1).

### *De novo* genome assembly

Reads used to construct *de novo* genome assemblies were error corrected with Quake v0.3.5, specifying default settings and a kmer length of 19 (ref. 60) (Supplementary Table 2). Optimal kmer length for *de novo* genome assemblies was estimated using kmergenie v1.6741 (ref. 61).

We constructed a female de novo genome assembly with DNA-seq reads from two females using SOAPdenovo v2.04 (ref. 62) and specifying the multi-kmer option with a starting kmer of 37 and max kmer of 55. All reads were used during both contig and scaffold assembly. During scaffolding (SOAPdenovo scaff), the –F parameter was set to specify that gaps in scaffolds should be filled. Lastly, GapCloser was used to close gaps emerging during the scaffolding process. Sequences<1 Kb in length were filtered from the assembly (Supplementary Table 3).

### Assigning chromosomal position

Guppy genes were downloaded from RefSeq (Guppy_female_1.0+MT, RefSeq assembly accession: GCF_000633615.1) and the longest isoform picked for each. Coding sequences were BLASTed against the *de novo* genome assembly using BLASTn v2.3.0 (ref. 63) with an e-value cutoff of 10e^−10^ and minimum percentage identity of 30%. When genes mapped to multiple locations, the top blast hit was chosen using the highest BLAST score.

*De novo* scaffolds were assigned to the guppy reference chromosomes and oriented using the chromosomal location and start position of mapped guppy genes. If multiple genes mapped to a given scaffold, the scaffold was assigned to the reference chromosome that the majority of genes were located on. Specifically, at least 70% of genes mapping to a given scaffold must be located on the same chromosome in the reference genome otherwise the scaffold was discarded. The degree of concordance in assigned chromosome position using this approach is high (Supplementary Table 4), and only 320 scaffolds from the female genome assembly were discarded due to discordance between chromosomal locations.

### Genomic coverage analysis

Male and female trimmed DNA-seq reads were separately mapped to the *de novo* genome assembly using Bwa v0.7.12 aln/sampe with default settings^64^. Uniquely mapped reads were extracted using grep ‘XT:A:U' and soap.coverage v2.7.9 (http://soap.genomics.org.cn) was used to extract coverage of scaffolds in every individual. For each scaffold, coverage was defined as the total number of times each site was sequenced divided by the number of sites that were sequenced.

For lab populations, average coverage values were calculated for females and males separately. We added 1 to each value to avoid infinitely high numbers associated with log_2_ 0. Male:female coverage was calculated for each scaffold as log_2_(average male coverage) – log_2_(average female coverage).

For upstream and downstream wild populations, coverage was estimated using Bwa v0.7.15 aln/sampe and the same pipeline as the lab populations. Average coverage was calculated for each gene separately across each population. To account for differences in sequencing depth across populations, the log_2_ coverage for each gene was normalized by the median log_2_ coverage of X chromosome (log_2_ coverage—median log_2_ coverage of X chromosome). Male:female coverage was estimated for each population relative to the normalized coverage of the female lab population.

### Polymorphism analysis

Male and female trimmed DNA-seq reads from both wild and lab populations were separately mapped to the *de novo* genome assembly using Bowtie1 v1.1.2 (ref. 65), specifying a maximum insert size for paired-end alignment of 1,400 and writing hits in map format. Map files were sorted by scaffold and bow2pro v0.1 (http://guanine.evolbio.mpg.de/) was used to generate a profile for each sample. Sites with coverage <10 were excluded from the analysis and SNPs were called when a site had a major allele frequency of 0.3 times the site coverage. SNPs were only included in further analyses if they were located within genic regions (see Expression analysis method for detail on gene annotation). Average SNP density for each gene was calculated as sum(SNPs) divided by sum(no. of filtered sites). We added 1 to each value to avoid infinitely high numbers associated with log_2_ 0. Genes were excluded if zero sites remained after filtering.

For lab populations, average SNP density was calculated separately for males and females. Male:female SNP density was calculated for each gene as log_2_(average male SNP density)−log_2_(average female SNP density).

For upstream and downstream wild populations, average SNP density was calculated for each gene separately across each population. To account for differences in overall genetic diversity across populations, the log_2_ SNP density for each gene was normalized by the median log_2_ SNP density of X chromosome (log_2_ SNP density—median log_2_ SNP density of X chromosome). Male:female SNP density was estimated for each population relative to the normalized SNP density of the female lab population.

To calculate the probability that the convergence in patterns of SNP density across populations we observe is due to chance, we randomly sampled 10 Mb windows across the autosomes 1,000 times. For each window, we tested whether the upstream normalized male:female SNP density was greater than the downstream population in each river using a one-tailed Wilcoxon ranked sum test. We looked for windows where all three rivers had *P*-values<0.05 and the median SNP density in the lab population was greater than the 95% autosomal confidence interval.

### Expression analysis

Male and female trimmed RNA-seq reads were separately mapped to the *de novo* genome assembly using HISAT2 v2.0.4 (ref. 66), suppressing unpaired and discordant alignments for paired reads and excluding reads from the sam output that failed to align. Reported alignments were tailored for transcript assemblers including StringTie.

Sam files were coordinate sorted using SAMtools v1.2 (ref. 67) and converted to bam files. StringTie v1.2.3 (ref. 67) was used to quantify gene expression and annotate the *de novo* assembly.

Specifically, StringTie was run on each sample with default settings and the output GTF files were merged. The combined GTF file was filtered to remove non-coding RNA (ncRNA) and transcripts less than 50 bp in length. Specifically, transcript sequences were extracted using bedtools getfasta^68^ and BLASTed to *Oryzias latipes* (MEDAKA1), *Gasterosteus aculeatus* (BROADS1), *Poecilia formosa* (PoeFor_5.1.2) and *Danio rerio* (GRCz10) ncRNA downloaded from Ensembl 84 (ref. 69). Transcripts with blast hits to ncRNA were removed from the GTF file. StringTie was rerun on each sample and expression was only estimated for genes defined in the filtered GTF file. A minimum expression threshold of 2FPKM in at least half of the individuals of either sex was imposed. This final filtered data set (23,603 genes) was used in subsequent expression and polymorphism analyses.

Expression was normalized using EdgeR^70^. Sam files were name sorted using SAMtools and HT-seq count v0.6.1 (ref. 71) used to extract read counts for each gene. Genes were excluded if they were not located on scaffolds assigned to the guppy reference genome. In all, 13,306 genes remained after filtering. Expression was normalized using TMM (trimmed mean of m-values) in EdgeR and RPKM estimated for each gene. Individuals cluster transcriptomically by sex (Supplementary Fig. 4). Average RPKM for each gene was calculated separately for males and females. We added 1 to each value to avoid infinitely high numbers associated with log_2_ 0. Male:female expression was calculated for each gene as log_2_ (average male RPKM)—log_2_(average female RPKM).

We tested whether there was an enrichment of GO terms in the X-Y diverged region compared with the rest of the genome. *Danio rerio* (GRCz10) coding sequences were downloaded from Ensembl 84 (ref. 69) and the longest isoform extracted for each gene. Longest isoforms were extracted for our set of expressed guppy genes and BLASTed to *D. rerio* using BLASTn v2.3.0 (ref. 63) with an e-value cutoff of 10e^−10^ and minimum percentage identity of 30%. When genes mapped to multiple locations, the top blast hit was chosen using the highest BLAST score. *D. rerio* orthologues were identified for genes in the X-Y degenerate region (15–25 Mb) and compared with the remainder of the genome using GOrilla^72^,^73^.

### Cluster analysis of expression data

Transcriptional similarity of normalized count data for female and male individuals was assessed using a multi-dimensional scaling plot (MDS) with default settings in EdgeR^74^. RPKM data was clustered using the R package pheatmap and boostrap values calculated using pvclust. UPGMA was used in the hierarchical cluster analysis and the distance matrix was computed using the Euclidean method.

### Calculating moving averages

Moving averages of coverage/polymorphism/expression were calculated in R^75^ based on sliding window analyses using the roll_mean function. Ninety-five per cent confidence intervals for the moving average were calculated by randomly resampling (1,000 times, without replacement) autosomal scaffolds (coverage analysis) or genes (SNP density and expression analyses).

### Faster-X analysis

Guppy transcript sequences were extracted using bedtools getfasta^68^ and the longest isoform chosen for each of the 23,603 genes. Genes on genomic scaffolds without chromosomal locations were removed, leaving 13,306 genic sequences for the Faster-X analysis. *Oryzias latipes* (MEDAKA1), *Xiphophorus maculatus* (Xipmac4.4.2), *Poecilia formosa* (PoeFor_5.1.2) were downloaded from Ensembl 84 (ref. 69) and the longest transcript for each gene was identified. We determined orthology using reciprocal BLASTn v2.3.0 (ref. 63) with an e-value cutoff of 10e^−10^ and minimum percentage identity of 30%. When genes mapped to multiple locations, the top blast hit was chosen using the highest BLAST score. In all, 7,382 reciprocal 1-1 orthologues across the four species were identified. We obtained open reading frames and protein coding sequence with BLASTx v2.3.0 with an e-value cutoff of 10e^−10^ and minimum percentage identity of 30% using the approach in Wright *et al*.^76^ Reciprocal orthologues with no BLASTx hits or a valid protein-coding sequence were excluded.

Reciprocal orthologues were aligned with PRANK v.140603 (ref. 77) using the codon model and specifying the following guidetree; (((*Poecilia reticulata*, *Poecilia formosa*), *Xiphophorus maculatus*), *Oryzias latipes*). SWAMP v 31-03-14 (ref. 78) was used to mask erroneous sequences in the alignments. Reciprocal orthologues were discarded if the alignment length was <300 bp after removing gaps and masked sites. After this length filter, 5,349 reciprocal orthologues remained.

We used the branch model (model=2, nssites=0) in the CODEML package in PAML v4.8 (ref. 79) to obtain divergence estimates using the following phylogeny; ((*Poecilia reticulata*, *Poecilia formosa*), *Xiphophorus maculatus*, *Oryzias latipes*). The branch model was used to calculate mean *d*_N_/*d*_S_ across the *Poecilia reticulata* branch. As mutational saturation and double hits can lead to inaccurate divergence estimates^80^ orthogroups were excluded if *d*_S_ >2.

Orthologues were divided into genomic categories on the basis of their chromosomal location. For each category, mean *d*_N_ and mean *d*_S_ were calculated as the sum of the number of substitutions across all orthologues divided by the number of sites (*d*_N_=sum *D*_N_/sum N, *d*_S_=sum *D*_S_/sum S, where *D*_N_ and *D*_S_ are estimates of the number of nonsynonymous or synonymous substitutions and N and S are the number of nonsynonymous/synonymous sites). This approach prevents disproportionate weighting of shorter genes by avoiding the problems of infinitely high *d*_N_/*d*_S_ estimates arising from sequences with extremely low *d*_S_ (refs 76, 81, 82).

Significant differences in *d*_N_, *d*_S_ and *d*_N_/*d*_S_ between genomic categories were determined using permutation tests with 1,000 replicates. One-tailed tests were used to test for the Faster-X effect where we predict *d*_N_/*d*_S_ is greater for X-linked gene relative to the autosomes. Two-tailed tests were used to test for differences in *d*_N_ and *d*_S_. Bootstrapping with 1,000 repetitions was used to generate 95% confidence intervals.

### Phylogenetic history of guppy populations

Using DNA-seq data, we reconstructed the phylogenetic relationships between the six wild populations. We mapped trimmed reads to the previously sequenced guppy genome (Guppy_female_1.0+MT, RefSeq assembly accession: GCF_000633615.1) using Stampy v1.0.28 (ref. 83) with a substitution rate of 0.01. After mapping, sam files were converted to bam and coordinate sorted using SAMtools v1.2 (ref. 84) and then deduplicated using Picard tools v1.136 (ref. 85). Subsequently, we added read groups and merged libraries belonging to the same individual using Picard. We then called variants on all 24 individuals simultaneously using two independent methods (GATK and Platypus), and retained only SNPs called reliably with both methods and passing quality control filters.

As part of the GATK variant calling pipeline, v3.4.46 (ref. 86), we first realigned reads around indels and recalibrated base quality scores. We then proceeded with variant calling using the HaplotypeCaller and GenotypeGVCFs tools. The second method we employed to call variants was Platypus v0.8.1 (ref. 87), which we ran in assembly mode, restricting calling to reads mapping to the 23 canonical chromosomes (that is, excluding those mapped to unplaced scaffolds).

After variant calling we removed indels, intersected the GATK and Platypus SNP sets, and applied stringent quality filtering. We removed singleton SNPs, multiallelic SNPs and SNPs failing the following quality thresholds: quality by depth>2, coverage>0.5x and<2x mean coverage, >2 reads for the alternative allele, mapping quality>40, allele bias *Z* score for mapping quality, base quality or read position<-1.96, or strand bias Fisher exact test *P*>0.05. We also removed SNPs with missing genotype in any individual. This yielded 4.6 million high-quality SNPs.

Next, we used R package adegenet v2.0.1 (ref. 88) to construct a Euclidian distance matrix for the 24 individuals based either on all SNPs across the genome or on only the 72,623 SNPs between 15 and 25 MB on the X chromosome. We used the R package ape v3.5 (ref. 89) to produce from each matrix a simple neighbour joining tree to visualize the genetic distance between the six populations, and performed 100 bootstrap iterations to assess support for each node.

### Data availability

RNA and DNA reads have been deposited at the NCBI Sequencing Read Archive, BioProject ID PRJNA353986.

## Additional information

**How to cite this article:** Wright, A. E. *et al*. Convergent recombination suppression suggests role of sexual selection in guppy sex chromosome formation. *Nat. Commun.* **8,** 14251 doi: 10.1038/ncomms14251 (2017).

**Publisher's note:** Springer Nature remains neutral with regard to jurisdictional claims in published maps and institutional affiliations.

## Supplementary Material

Supplementary Information Supplementary Figures and Supplementary Tables
