# Supplementary material for: Convergent recombination suppression suggests role of sexual selection in guppy sex chromosome formation
Source: Nat Commun. 2017 Jan 31;8:14251. doi: 10.1038/ncomms14251 (PMC5290318; doi:10.1038/ncomms14251)
Supplement: Supplementary Information — Supplementary Figures and Supplementary Tables [file ncomms14251-s1.pdf]

Supplementary Table 1: Sequencing information for each sample

Sample	Source	Method	Coverage	Raw paired reads	Paired reads after trimming	% removed
Female_4	Lab pop	DNA-seq	79X	276,700,698	253,668,178	8.32
Female_7	Lab pop	DNA-seq	75X	262,392,382	233,869,784	10.87
Male_11	Lab pop	DNA-seq	68X	238,377,854	217,155,394	8.90
Male_8	Lab pop	DNA-seq	85X	298,121,974	271,223,610	9.02
Female_1	Lab pop	RNA-seq	-	31,614,139	30,942,700	2.12
Female_4	Lab pop	RNA-seq	-	42,162,172	41,258,427	2.14
Female_7	Lab pop	RNA-seq	-	29,059,783	28,520,214	1.86
Female_13	Lab pop	RNA-seq	-	32,392,057	31,628,760	2.36
Male_5	Lab pop	RNA-seq	-	26,029,449	25,434,019	2.29
Male_8	Lab pop	RNA-seq	-	31,955,977	31,182,426	2.42
Male_11	Lab pop	RNA-seq	-	36,271,770	35,430,424	2.32
Male_17	Lab pop	RNA-seq	-	30,579,016	29,853,906	2.37
Male_6	Lab pop	RNA-seq	-	31,303,333	30,559,027	2.38
Male_9	Lab pop	RNA-seq	-	37,546,710	36,718,647	2.21
Male_15	Lab pop	RNA-seq	-	29,875,402	29,258,994	2.06
Male_18	Lab pop	RNA-seq	-	39,603,241	38,785,961	2.06
Male_12	Lab pop	RNA-seq	-	32,568,129	31,793,985	2.38
Male_2	Lab pop	RNA-seq	-	23,196,956	22,777,671	1.81
Male_14	Lab pop	RNA-seq	-	31,859,225	31,110,939	2.35
Male_1	Wild AD	DNA-seq	37X	130,231,185	118,932,936	8.68
Male_3	Wild AD	DNA-seq	29X	101,814,846	93,738,591	7.93
Male_4	Wild AD	DNA-seq	30X	104,256,689	96,147,514	7.78
Male_5	Wild AD	DNA-seq	41X	1438,00,633	131,847,549	8.31
Male_12	Wild YU	DNA-seq	36X	126,989,240	114,251,020	10.03
Male_13	Wild YU	DNA-seq	40X	140,058,828	129,438,072	7.58
Male_14	Wild YU	DNA-seq	30X	103,948,475	95,917,845	7.73
Male_15	Wild YU	DNA-seq	29X	102,834,035	93,981,379	8.61
Male_16	Wild QD	DNA-seq	36X	126,993,935	115,367,896	9.15
Male_17	Wild QD	DNA-seq	33X	117,037,503	107,793,725	7.90
Male_19	Wild QD	DNA-seq	37X	129,116,621	118,530,011	8.20
Male_20	Wild QD	DNA-seq	30X	105,910,625	97,094,279	8.32
Male_32	Wild AU	DNA-seq	38X	133,405,487	121,078,915	9.24
Male_33	Wild AU	DNA-seq	36X	126,870,348	116,645,902	8.06
Male_34	Wild AU	DNA-seq	34X	120,555,304	111,191,193	7.77
Male_35	Wild AU	DNA-seq	33X	116,057,513	105,723,853	8.90
Male_41	Wild YD	DNA-seq	40X	139,172,741	127,519,332	8.37
Male_42	Wild YD	DNA-seq	37X	129,566,748	119,662,352	7.64
Male_43	Wild YD	DNA-seq	27X	94,884,948	87,529,008	7.75
Male_44	Wild YD	DNA-seq	30X	103,984,825	94,569,988	9.05
Male_51	Wild QU	DNA-seq	28X	98,581,145	89,880,179	8.83
Male_52	Wild QU	DNA-seq	36X	125,273,377	112,674,653	10.06
Male_53	Wild QU	DNA-seq	31X	107,398,826	99,357,018	7.49

Male_54	Wild QU	DNA-seq	46X	160,156,505	147,797,565	7.72

A = Aripo watershed, Y = Yarra watershed, Q = Quare watershed
U = Upstream population, D = Downstream population

Supplementary Table 2: Error corrected reads for de novo genome assembly

Sex	Source	Sequencing	No. of individuals	Coverage	Error corrected paired reads
Female	Lab pop	DNA-seq	2	137X	479,641,121

Supplementary Table 3: Female de novo genome assembly statistics

Pre 1kb length filter				Post 1kb length filter				Oriented scaffolds			
No.	N50 (Kb)	N90 (Kb)	Length (Mb)	No.	N50 (Kb)	N90 (bp)	Length (Mb)	No.	N50 (Kb)	N90 (Kb)	Length (Mb)
2,361,160	5.4	0.1	963.6	96,611	11.3	2.8	634.8	19,206	17.4	5.6	219.5

Supplementary Table 4: Assignment of chromosomal position

Reference genome	Genes	Genes mapped to assembly	Scaffolds with mapped genes	Scaffolds discarded due to mapping discordance	Oriented scaffolds	% with multiple mapped genes all from the same reference chromosome
Guppy	25,694	25,460	19,526	320	19,206	92%

Supplementary Table 5: Coverage and SNP density

	Autosomes				X chromosome			
	No.	M log_2 median	F log_2 median	M:F log_2 median	No.	M log_2 median	F log_2 median	M:F log_2 median
**Coverage** (Wilcoxon rank sum test p-value)	17,353	5.8505	5.8477	0.0024	709	5.8273 (0.0224)	5.8324 (< 0.001)	-0.0115 (<0.001)
**SNP density** (Wilcoxon rank sum test p-value)	13,286	0.0023	0.0022	0.0000	555	0.0030 (<0.001)	0.0023 (0.837)	0.0007 (<0.001)

Wilcoxon rank sum test between autosomal and X chromosome medians

Supplementary Table 6: Faster-X Effect

	Autosomes	X-Y diverged region	Stratum II	Stratum I
**No.**	4755	86	70	16
**d_N/d_S**	0.091	0.107	0.105	0.106
(95% CI)	0.088-0.094	0.088-0.129	0.084-0.131	0.067-0.172
(p-value)		0.067	0.107	0.249
**d_N**	0.003	0.004	0.004	0.006
(95% CI)	0.003-0.003	0.003-0.005	0.003-0.004	0.004-0.009
(p-value)		**0.012**	0.182	**0.002**
**d_S**	0.034	0.037	0.034	0.057
(95% CI)	0.033-0.034	0.033-0.042	0.030-0.039	0.043-0.069
(p-value)		0.1	0.77	**<0.001**

X-Y diverged region refers to Stratum II (15-22Mb) & Stratum I (> 22Mb)
P-values calculated relative to the autosomes using permutation tests with 1000 replicates. Confidence intervals calculated using bootstrapping with 1000 replicates.

Supplementary Table 7: Normalised SNP densities in the X-Y diverged region across upstream and downstream guppy populations

	Downstream			Upstream		
**River**	**No.**	**M $\log_2$ median**	**M:F $\log_2$ median**	**No.**	**M $\log_2$ median**	**M:F $\log_2$ median**
**Yarra**	197	-0.0001	0.0006	194	0.0006	0.0013
(Wilcoxon rank sum test p-value)					(0.0002)	(0.0105)
**Quare**	196	-0.0001	0.0006	196	0.0001	0.0009
(Wilcoxon rank sum test p-value)					(0.0090)	(0.0342)
**Aripo**	195	-0.0000	0.0007	197	0.0002	0.0013
(Wilcoxon rank sum test p-value)					(0.0039)	(0.0209)

X-Y diverged region refers to Stratum II (15-22Mb) & Stratum I (> 22Mb)
Wilcoxon rank sum test between downstream and upstream medians

Supplementary Table 8: Normalised coverage in Stratum I of the sex chromosome across upstream and downstream guppy populations

	Downstream			Upstream		
**River**	**No.**	**M log_2 median**	**M:F log_2 median**	**No.**	**M log_2 median**	**M:F log_2 median**
**Yarra** (Wilcoxon rank sum test p-value)	59	0.0183	-0.0484	59	0.0065 (0.7345)	-0.0330 (0.8548)
**Quare** (Wilcoxon rank sum test p-value)	59	0.0365	-0.0289	59	0.0037 (0.5011)	-0.0444 (0.2494)
**Aripo** (Wilcoxon rank sum test p-value)	59	0.0292	-0.0519	59	0.0241 (0.6129)	-0.0329 (0.9314)

Wilcoxon rank sum test between downstream and upstream medians

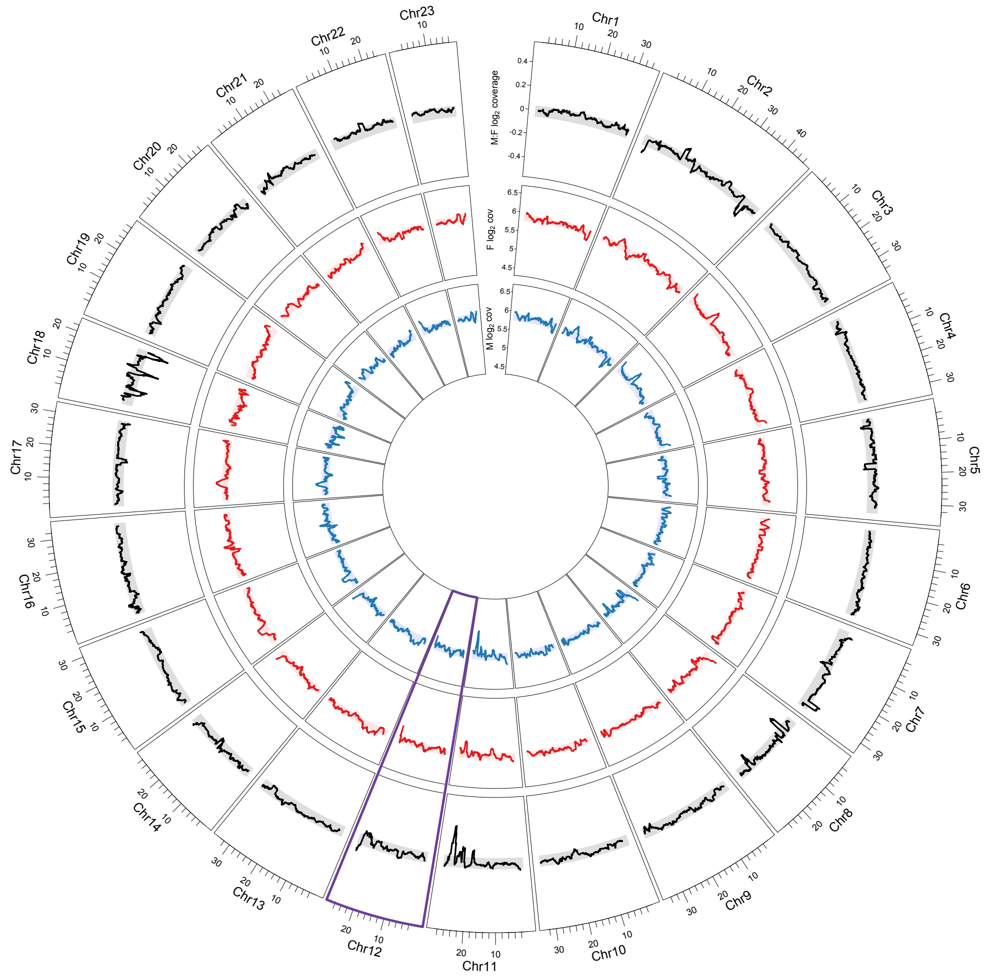

Supplementary Fig. 1. Circos plot of male and female coverage for oriented scaffolds, with moving averages based on window sizes of 40 scaffolds. For each chromosome, male coverage (blue), female coverage (red) and male:female coverage (black) is shown. 95% confidence intervals based on bootstrapping autosomal estimates are shown for male coverage (light blue), female coverage (pink) and male:female coverage (grey). The X chromosome, which contains the sex determining gene, is highlighted in purple.

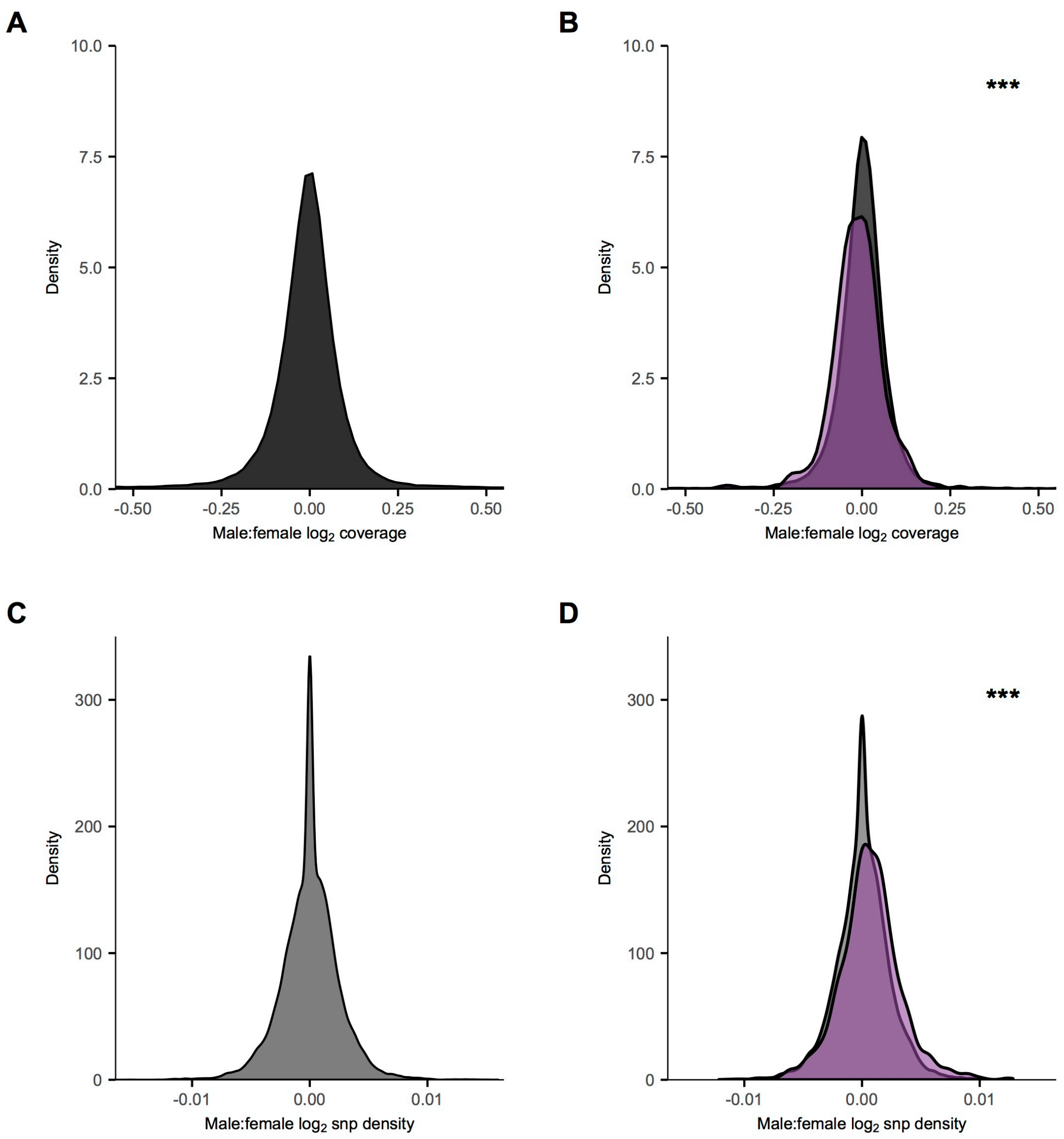

Supplementary Fig. 2. Distribution of male:female coverage (A and B) and SNP density (C and D) for assembled scaffolds. Panel A. Coverage differences for all scaffolds greater than 1Kb in length. Panel. B. Coverage differences for autosomal (grey) and the X chromosome (purple) scaffolds. Panel C. Differences in male and female SNP density for all scaffolds greater than 1Kb in length. Panel D. Differences in male and female SNP density for autosomes (grey) and the X chromosome (purple). *** p-value < 0.001.

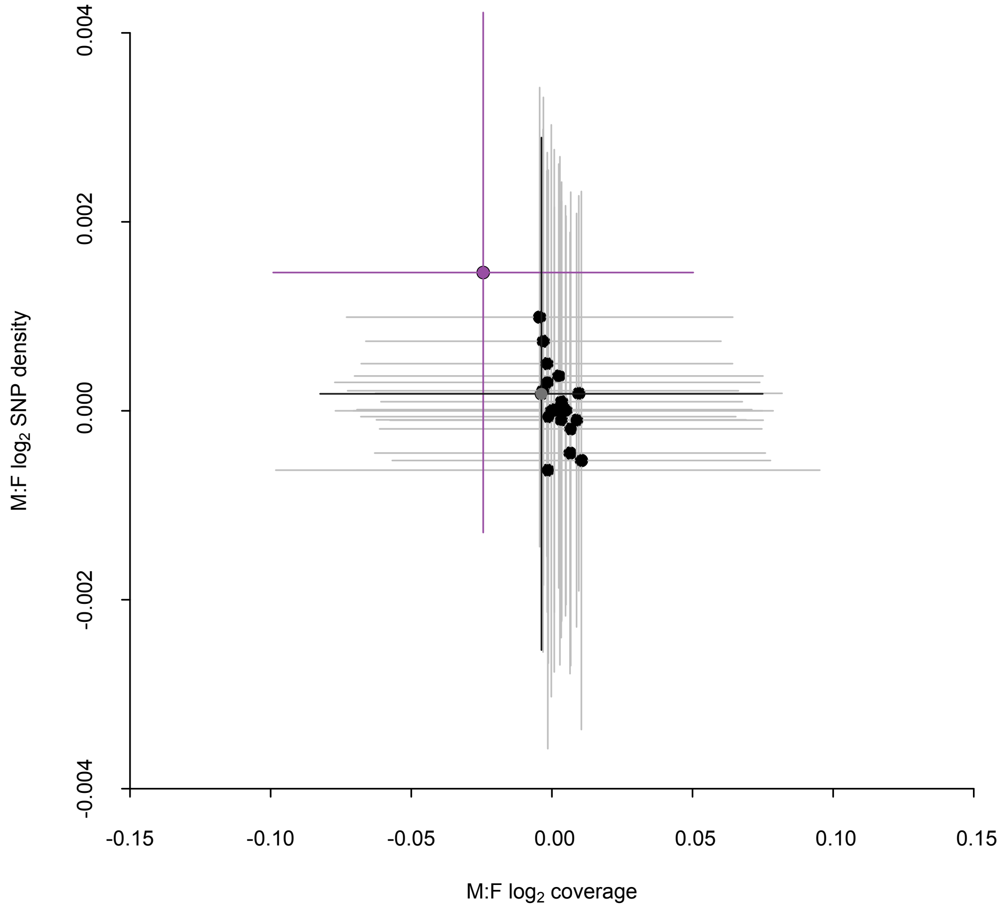

Supplementary Fig. 3. Distribution of sex differences in coverage and SNP density for all chromosomes. The X-Y diverged region (Strata I & II, 15 – 25 Mb) is in purple and PAR (<15 Mb) is in grey. Horizontal and vertical lines denote interquartile ranges.

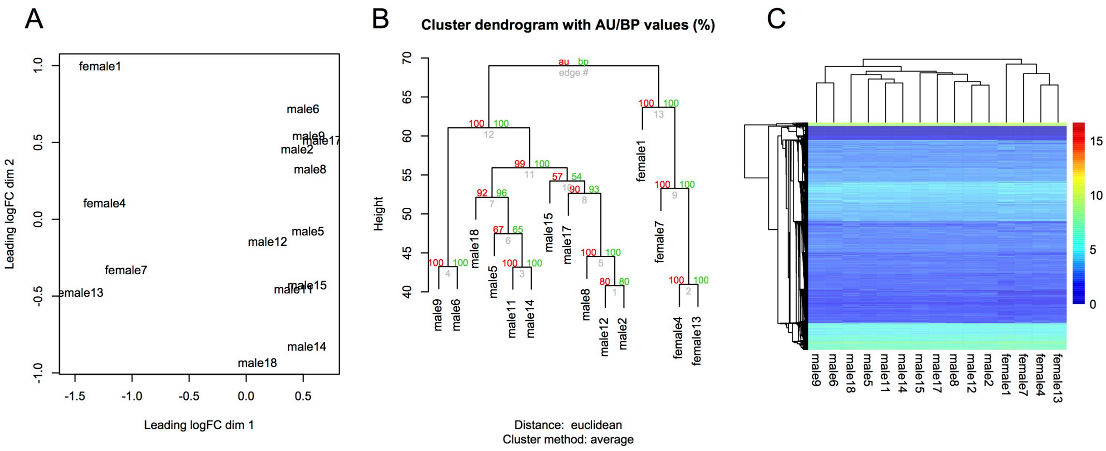

Supplementary Fig. 4. Cluster analysis of expression data. Panel A. MDS plot of normalized count data. Panel B. Cluster dendrogram of normalized $\log_2$ RPKM values. Approximately unbiased p-values are shown in red and bootstrap probability values are shown in green. Panel C. Clustered heatmap of normalized $\log_2$ RPKM values.

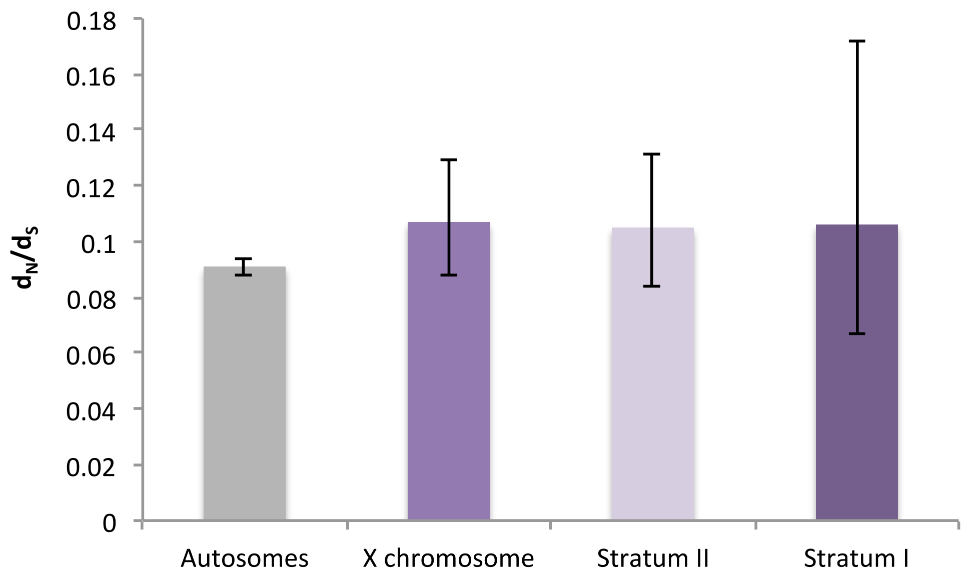

Supplementary Fig. 5. Estimates of mean d_N/d_S for the autosomes and the X chromosome. 95% confidence intervals were calculated by bootstrapping with 1000 replicates. X chromosome refers only to the X-Y region (Strata I & II, 15 – 25 Mb) and does not include genes in the PAR.

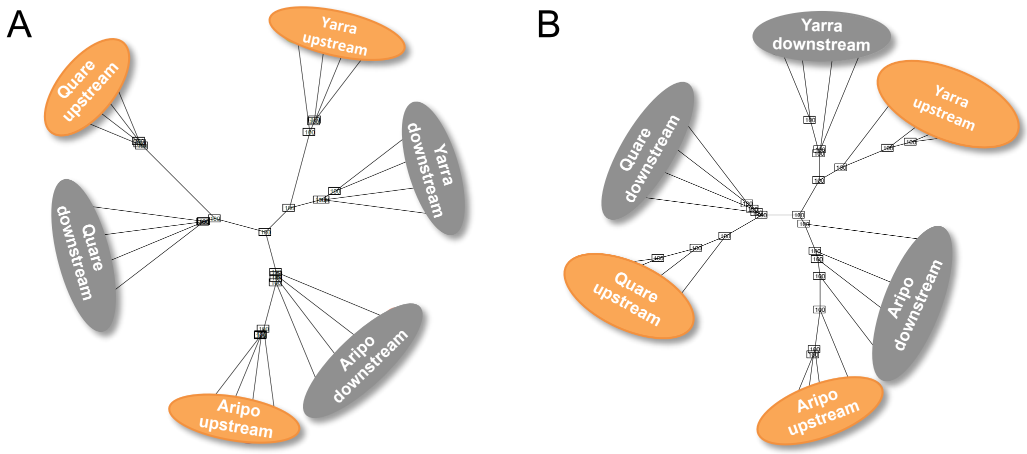

Supplementary Fig. 6. Phylogeny of upstream (orange) and downstream (grey) guppy populations across three watersheds (Yarra, Quare, Aripo) in Trinidad based on (A) all 4.6 million SNPs across the whole genome or (B) 72,623 SNPs located on the X-Y diverged region (Strata I & II, 15 – 25 Mb). Neighbor joining trees were constructed from Euclidian distances between individuals. Bootstrap support is indicated at each node.

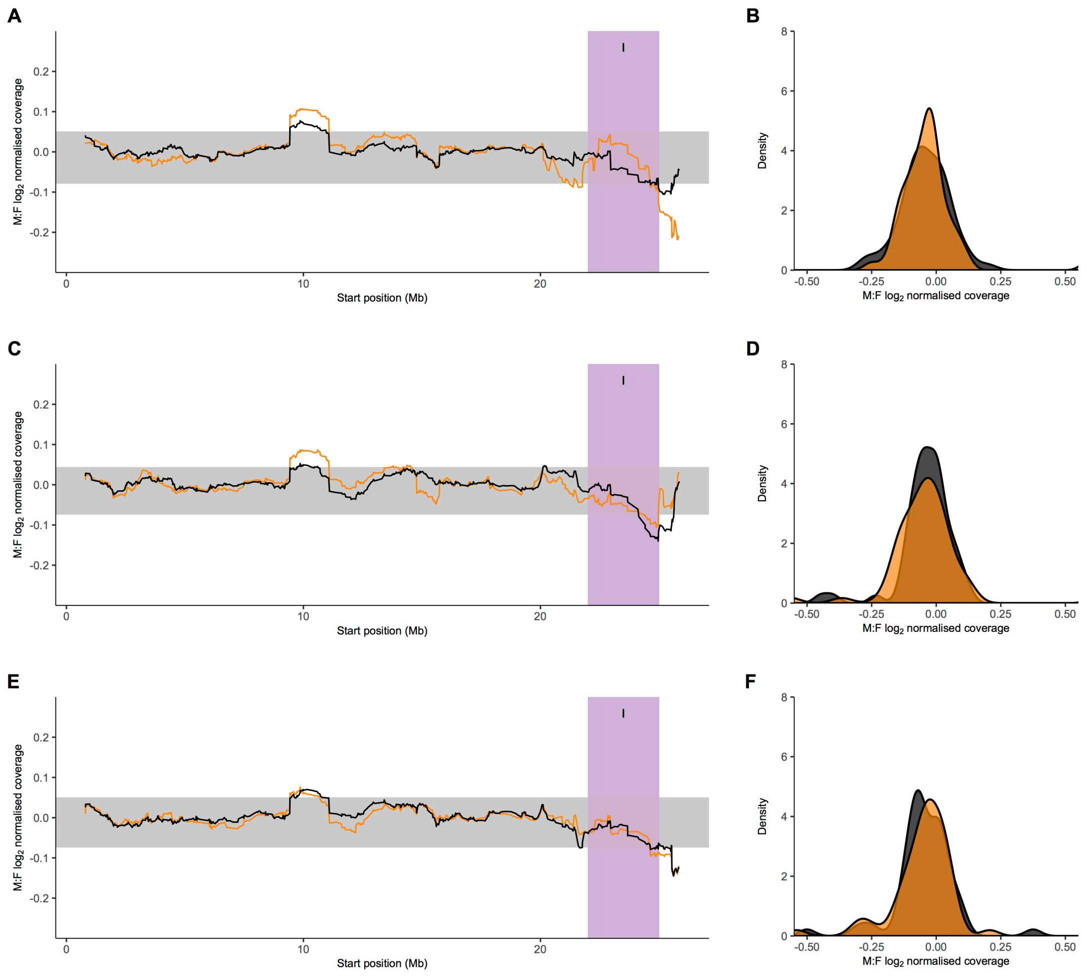

Supplementary Fig. 7. Male:female coverage for the X chromosome across upstream (orange) and downstream (black) guppy populations. Panels A. C. and E. Moving averages of normalised coverage across the X chromosome based on sliding window analysis (window size of 40 scaffolds) for Yarra (panel A), Quare (panel C) and Aripo (panel E) watersheds. 95% confidence intervals based on bootstrapping autosomal estimates are in grey. Dark purple indicates the region of the sex chromosomes with the greatest X-Y sequence divergence, where coverage is significantly less in laboratory population males (Stratum I, 22-25 Mb) (see Fig. 2), light purple indicates the region with less X-Y differentiation, where there a significant excess of male SNPs in laboratory populations (Stratum II, 15 – 22 Mb). Panels B. D. and F. Distribution of sex differences in normalised coverage for the oldest region of the X chromosome (Stratum I, 22 – 25 Mb) for Yarra (panel B), Quare (panel D) and Aripo (panel F) watershed.
